# The Evolving Role of Monomethyl Fumarate Treatment as Pharmacotherapy for Relapsing-Remitting Multiple Sclerosis

**DOI:** 10.7759/cureus.57714

**Published:** 2024-04-06

**Authors:** Alan D Kaye, John Lacey, Viet Le, Ahmed Fazal, Nicole A Boggio, Dorothy H Askins, Lillian Anderson, Christopher L Robinson, Antonella Paladini, Chizoba N Mosieri, Adam M Kaye, Shahab Ahmadzadeh, Sahar Shekoohi, Giustino Varrassi

**Affiliations:** 1 Department of Anesthesiology, Louisiana State University Health Sciences Center, Shreveport, USA; 2 School of Medicine, Louisiana State University Health Sciences Center, New Orleans, USA; 3 School of Medicine, Tulane University, New Orleans, USA; 4 Department of Anesthesiology, Tulane University, New Orleans, USA; 5 Department of Anesthesiology, Critical Care and Pain Medicine, Beth Israel Deaconess Medical Center, Harvard Medical School, Boston, USA; 6 Department of Life, Health, and Environmental Sciences (MESVA), University of L'Aquila, L'Aquila, ITA; 7 Department of Pharmacy Practice, Thomas J. Long School of Pharmacy and Health Sciences, University of the Pacific, Stockton, USA; 8 Department of Pain Medicine, Paolo Procacci Foundation, Rome, ITA

**Keywords:** autoimmune disease, disease-modifying therapy, multiple sclerosis, bafiertam®, monomethyl fumarate

## Abstract

Multiple sclerosis is the most common autoimmune disease affecting the central nervous system (CNS) worldwide. Multiple sclerosis involves inflammatory demyelination of nerve fibers in the CNS, often presenting with recurrent episodes of focal sensory or motor deficits associated with the region of the CNS affected. The prevalence of this disease has increased rapidly over the last decade. Despite the approval of many new pharmaceutical therapies in the past 20 years, there remains a growing need for alternative therapies to manage the course of this disease. Treatments are separated into two main categories: management of acute flare versus long-term prevention of flares via disease-modifying therapy. Primary drug therapies for acute flare include corticosteroids to limit inflammation and symptomatic management, depending on symptoms. Several different drugs have been recently approved for use in modifying the course of the disease, including a group of medications known as fumarates (e.g., dimethyl fumarate, diroximel fumarate, monomethyl fumarate) that have been shown to be efficacious and relatively safe. In the present investigation, we review available evidence focused on monomethyl fumarate, also known as Bafiertam®, along with bioequivalent fumarates for the long-term treatment of relapsing-remitting multiple sclerosis.

## Introduction and background

Multiple sclerosis (MS) is the most common autoimmune disease affecting the central nervous system (CNS), and it causes inflammatory demyelination of nerve fibers, resulting in focal sensory and/or motor deficits associated with the region of the brain/spinal cord affected. In 2020, it was estimated that 2.8 million people were affected by MS worldwide, a number that is growing quickly. In countries that reported prevalence data, global prevalence increased by 50% from 29 per 100,000 in 2013 to 44 per 100,000 in 2020 [1,2]. There are nearly one million cases in the United States, at a prevalence rate of 288 per 100,000, with more than double that concentration in females aged 45-64 [3]. US women are 2.8 times more likely to develop MS than men [3-6]. Some of the most common presentations of MS include optic neuritis, resulting in gradual unilateral vision loss; transverse myelitis, resulting in progressive pain, weakness, and sensory changes below the level of the lesion; internuclear ophthalmoplegia, resulting in diplopia; and gradual onset sensory loss or motor weakness on one side of the face [1].

MS is further subcategorized based on disease progress and recurrence of symptoms, with subtypes including clinically isolated syndrome (CIS), radiologically isolated syndrome (RIS), primary progressive multiple sclerosis (PPMS), relapsing-remitting multiple sclerosis (RRMS), and secondary progressive multiple sclerosis (SPMS). CIS patients have unifocal or multifocal symptoms, which last at least 24 hours without evidence of infection; RIS patients show radiologic evidence of disease only; RRMS patients experience periods of active symptoms that either cease and remit or actively worsen [7]. SPMS is a sequela of RRMS, in which symptoms worsen and remissions become shorter and less frequent. Criteria for PPMS state that there must be evidence for at least one year of worsening disease along with either T2 MRI showing dissemination in space (DIS) or oligoclonal bands found in the cerebrospinal fluid [8]. PPMS is more often diagnosed retrospectively, with an amassing of symptoms in its advancement. This subtype has a prevalence of about 15% among MS patients [7,9]. The most common subtype by far is RRMS, which is characterized by distinct episodes, or flares, of symptoms with complete or incomplete recovery and is present in 60-85% of those diagnosed [1,9].

A wide array of disease-modifying therapy (DMT) has been developed for relapsing forms of MS. In the treatment of RRMS, early intervention is emphasized due to the potential to slow the disease in its inflammatory stages and thereby delay the neurodegenerative phase. Early interferon-beta (IFN-β) use benefits cognitive tests like the Paced Auditory Serial Addition Test (PASAT) and imaging such as T2 MRI [10]. Dimethyl fumarate (DMF) was approved by the Food and Drug Administration (FDA) as an oral drug for MS in 2013, followed by bioequivalents diroximel fumarate (DRF) in 2019 and monomethyl fumarate (MMF) in 2020. Because these fumarates are all bioequivalent to each other, they are believed to have essentially identical pharmacological profiles for efficacy and safety built from evidence gathered from the original studies of DMF, with the main differences between the three lying in dosing and adverse side effects [5,6].

In this review, therefore, we evaluate different classes of DMT for RRMS, evidence of MMF efficacy/treatment profile, and comparisons to similar drugs. This literature review was performed in 2023 via a wide-ranging online search for English-language studies concerning MMF in the treatment of MS, including databases PubMed, Cochrane Database of Systematic Reviews, the National Center for Biotechnology Information, ScienceDirect, and Google Scholar. Priority was placed on recent articles/data published within the last five years and primary manuscripts for use when possible. Keywords searched for included the following in various combinations: monomethyl fumarate, MMF, Bafiertam®, multiple sclerosis, MS, disease-modifying therapy, disease-modifying antirheumatic drugs, and DMARDS.

## Review

Overview of RRMS DMT categories

IFN-β, a low-efficacy medication, is administered intramuscularly or subcutaneously for RRMS. This drug improves the course of MS by upregulating CD73, which converts adenosine monophosphate (AMP) to adenosine, an anti-inflammatory agent that helps prevent damage to MS-afflicted areas; it may be used as the first-line treatment for RRMS, active SPMS, and CIS. Adverse side effects include flu-like symptoms like muscle aches, fever or chills, back pain, autoimmune thyroiditis, and rarely seizures [11,12]. Although administered orally, teriflunomide is also considered a low-efficacy DMT for RRMS. Its mechanism of action is by inhibiting pyrimidine synthesis in B and T lymphocytes, which slows their proliferation. Adverse side effects include teratogenicity, hepatotoxicity, pancreatic fibrosis, myelotoxicity, and peripheral neuropathy [11,13]. Glatiramer, a low-efficacy medication, is administered subcutaneously and is approved as the first-line therapy for RRMS and CIS. Its mechanism of action in MS works via the inhibition of T-cell receptors. Its primary adverse side effect is cutaneous necrosis [11,14]. Fumarates, intermediate-efficacy medications, are administered orally. The mode of action of these medications is causing lymphocytic apoptosis and inducing antioxidant effects in the CNS via nuclear factor (erythroid-derived 2)-like 2 (Nrf-2). Side effects include gastrointestinal (GI) issues and skin flushing [15,16]. High-efficacy medications include monoclonal antibodies such as natalizumab, which is administered intravenously and acts by blocking lymphocyte transport across the blood-brain barrier. They can be used as the second-line treatment for RRMS. The side effects of this medication include infection by the John Cunningham (JC) virus, leading to progressive multifocal leukoencephalopathy (PML), hepatotoxicity, and increased risk of other infections (Table 1) [11,17].

**Table 1:** Summarizing DMTs in the overview IFN-β: interferon-beta; DMT: disease-modifying therapy; AMP: adenosine monophosphate; RRMS: relapsing-remitting multiple sclerosis; SPMS: secondary progressive multiple sclerosis; CIS: clinically isolated syndrome; JC: John Cunningham; PML: progressive multifocal leukoencephalopathy; Nrf-2: nuclear factor (erythroid-derived 2)-like 2

Medication	Route of administration	Mechanism of action	Approved for	Efficacy	Adverse side effects
IFN-β	Intramuscular or subcutaneous	Upregulates CD73, converting AMP to adenosine, an anti-inflammatory agent	RRMS, SPMS, CIS	Low	Flu-like symptoms, muscle aches, fever, chills, back pain, autoimmune thyroiditis, seizures (rarely)
Teriflunomide	Oral	Inhibits pyrimidine synthesis in B and T lymphocytes; slows proliferation	RRMS	Low	Teratogenicity, hepatotoxicity, pancreatic fibrosis, myelotoxicity, peripheral neuropathy
Glatiramer	Subcutaneous	Inhibits T-cell receptors	RRMS, CIS	Low	Cutaneous necrosis
Fumarates	Oral	Cause lymphocytic apoptosis; induce antioxidant effects via Nrf-2	RRMS	Intermediate	Gastrointestinal issues, skin flushing
Natalizumab	Intravenous	Blocks lymphocyte transport across the blood-brain barrier	RRMS (second-line)	High	Infection by the JC virus leading to PML, hepatotoxicity, and increased risk of other infections

Role of fumarates

Fumarates, including DMF, DRF, and MMF, are oral therapies for treating MS in adults. They are part of the DMT options available for RRMS, which aim to reduce relapse rates, slow down the accumulation of brain lesions, and decrease inflammation and injury in the brain. Fumarates are preferred for patients who value self-administered oral medications over injections or infusions. When considering DMT options for RRMS, the choice of therapy depends on several factors, including the patient's preferences, drug-specific factors, and risk assessments. Fumarates have intermediate efficacy compared to other DMTs, such as monoclonal antibodies or S1P receptor modulators, but they are still effective in reducing relapse rates and slowing down disease progression [18]. Treatment decisions should be made through shared decision-making between the patient and their healthcare provider, considering the patient's values, preferences, and specific clinical characteristics. Before initiating fumarate therapy or any other DMT, patients should undergo baseline assessments and immunization reviews to ensure they are up to date with vaccinations. Regular monitoring of clinical parameters and laboratory tests is also required during treatment to assess the patient's response and monitor for any potential side effects. Overall, fumarates play a valuable role as an oral DMT option for adults with RRMS, providing convenience and effectiveness in managing the disease [19].

MMF

MMF, also known as Bafiertam®, is an approved oral treatment by the US FDA for MS in adults, including active SPMS, RRMS, and CIS [20]. Approved in April 2020, MMF is the sole active metabolite of DMF and DRF, two drugs already widely used in treating MS.

Mechanism of Action

Though MMF's mechanism of action in treating the pathophysiology of MS has not yet been fully elucidated, MMF has been known to interact with various immunomodulatory nuclear transcription factor pathways. Treatment of primary cell cultures and animals with DMF has been shown to increase levels of active Nrf-2 in the CNS [20]. The mechanism of action involves, initially, the binding of the unsaturated carboxylic acid esters in MMF to glutathione (GSH), which leads to sub-toxic concentrations of antioxidants, facilitating a reactive response by which Nrf-2 is activated [21]. When activated, Nrf-2 responds by enacting the transcription of antioxidative proteins such as GSH-producing enzymes and heme oxygenase-1 (HO-1) [22]. This is supported by studies that show that MMF-treated mice with knock-outs of Nrf-2 were not found to have upregulated amounts of upregulated antioxidant target genes [20]. MMF has additionally been shown to be a strong agonist of the hydroxycarboxylic acid receptor 2 (HCAR2), a G-protein-coupled receptor found on myelocytes such as neutrophils, dendritic cells, and macrophages [23]. The binding of HCAR2's endogenous ligand in niacin has been shown to reduce neuroinflammation via the inhibition of NF-kB signaling, which may contribute to the anti-inflammatory effects of MMF in MS [24].

In addition to stimulating anti-inflammatory pathways, MMF treatment works to decrease the activation of pro-inflammatory pathways. In lipopolysaccharide-treated cell cultures of cerebral cortices prepared from rats, for example, astrocytes treated with DMF were shown to have decreased synthesis of pro-inflammatory mediators of nitrogen-oxide synthase, tumor necrosis factor-alpha (TNF-α), interleukin-1 beta (IL-1β), and interleukin-6 (IL-6) [25]. Fumarates have generally been shown to shift peripheral cell profiles in the immune system toward anti-inflammatory responses. Although these recorded changes occur peripherally and not in the CNS, a general goal of the treatment of MS is to decrease pro-inflammatory leukocytes being recruited through the blood-brain barrier [26]. DMF leads to a dose-dependent decrease in Bcl-2 expression, promoting lymphocyte apoptosis [27]. T lymphocytes are mainly influenced by fumarate-induced leukopenia, with CD8+ T cells experiencing a greater decrease in cell count than CD4+ T cells [28]. More notable than this, though, is the fact that there is a change in the relative distribution of T cells about their inflammatory status, as increased Th2/Th1Th17 and Treg/Th1Th17 ratios are indicative of an anti-inflammatory shift in these patients [4]. In addition to these peripheral effects, DMF has also been shown to decrease the transendothelial migration of immune cells, another potential source of benefit in treatment.

Undesired Side Effects

Common side effects of MMF use include flushing and GI symptoms. Clinical trials of DMF as its prodrug show that 40% (vs 6% placebo (PBO)) of patients experienced flushing associated with redness, warmth, and itching/burning sensation [28]. There is a range of intensity of this symptom, with ~3% discontinuing the drug due to flushing and less than 1% requiring hospitalization; it has also been shown to reduce incidence after aspirin administration [28]. GI symptoms such as abdominal pain (18% DMF, 10% PBO), diarrhea (14% DMF, 11% PBO), nausea (12% DMF, 9% PBO), and vomiting (9% DMF, 5% PBO) are the second most common response regarding fumarate treatment. GI events occurred most frequently in the first month of treatment, and approximately 4% of patients discontinued DMF related to GI events [28]. MMF's role in activating HCAR2-mediated signaling pathways has been linked to these symptoms, as even though HCAR2 has been linked to anti-inflammatory effects, pleiotropic activation of HCAR2 on different immune cells may lead to pro-inflammatory impacts, particularly in the GI system and skin [29]. It has been shown, for example, that the stimulation of HAR2 by MMF induces the cyclooxygenase-2 (COX-2)-mediated synthesis of prostaglandin in keratinocytes and Langerhans cells, leading to the skin flushing seen in treatment with MMF [30]. Lymphopenia has been a noted side effect of fumarate treatment ever since they were utilized in the treatment of psoriasis back in the 1950s [31]. On average, mean lymphocyte counts decrease by 30% during the first year of treatment and remain stable afterward [28]. Pro-apoptotic and anti-proliferative effects in lymphocytes such as T cells likely contribute to the therapeutic effect of reducing inflammation in MS. However, as a result, DMF clinical trials have reported increased susceptibility to opportunistic infections. PML, in particular, is an opportunistic viral infection of the brain, which has been seen more commonly in patients with more profound lymphopenia and may quickly lead to death [32,33]. Other potential opportunistic infections seen with DMF treatment include herpes zoster, tuberculosis, aspergillus, and cytomegalovirus. Less common but more serious reactions include anaphylaxis, angioedema, and hepatotoxicity [28]. As a result, these symptoms should be monitored, and regular liver function tests should be assessed. The onset of liver injury typically ranges from days to months after initiation in trials regarding DMF. At maximum elevation, one can expect the elevation of serum transaminases to be five times the normal limit. No reported cases of liver injury in trials resulted in liver failure or death [28]. Adverse effects in trials comparing DMF to PBO are mentioned in Table 2.

**Table 2:** Comparison of populations, results, and outcomes of DMF clinical trials DMF: dimethyl fumarate; BID: twice daily; TID: thrice daily; PBO: placebo; GA: glatiramer acetate; FTY: fingolimod; GI: gastrointestinal; TRF: teriflunomide

Clinical trial	Patient population	Efficacy results	Additional outcomes
DMF: 240 mg BID, DMF 240 mg TID, PBO [34]	n=1234 (DMF 240 mg BID, DMF 240 mg TID, PBO)	Reduced relapse rate by two years (27% DMF BID, 26% DMF TID, 46% PBO). Decreased disability progression (16% DMF BID, 18% DMF TID, 27% PBO). Decreased MRI lesions (P<0.001 versus PBO). Decreased annualized relapse rate (0.17 DMF BID, 0.19 DMF TID, 0.36 PBO)	Serious adverse events were similar across all study groups. Increased lymphopenia and liver aminotransferase levels
CONFIRM [35]	n=1417 (DMF 240 mg BID, DMF 240 mg TID, GA 20 mg, PBO)	Nonsignificant trends in disability progression (21% DMF BID, 24% DMF TID, 7% GA). Decreased MRI lesions (P<0.001 DMF BID, P<0.001 DMF TID, P=0.002 GA versus PBO). Decreased annualized relapse rate (0.22 DMF BID, 0.20 DMF TID, 0.29 GA, 0.40 PBO)	Adverse events such as flushing and GI events occurred at a higher rate with active treatment vs PBO. Noted decreased lymphocyte counts with DMF
ENDORSE [36]	n=1736 from DEFINE, ENDORSE participants (DMF 240 mg BID or PBO for 0-2 years; both groups received DMF 240 BID thereafter)	Annualized relapse rate of DMF to DMF remained low at 0.143 (0.120-0.169). Annualized relapse rate of PBO to DMF decreased after initiation and remained low throughout at 0.330 (0.266-0.408) 0-2 years and overall 0.151 (0.118-0.194). The majority of participants had no 24-week confirmed disability worsening over 10 years (72% DMF/DMF, 73% PBO/DMF)	Extension of DEFINE, CONFIRM followed for 13 years with DMF. 32% experience serious adverse events (mostly multiple sclerosis relapse). 14% discontinued treatments due to adverse events (4% GI disorders)
Hersh et al., 2017 [37]	n=775 (DMF n=458, FTY n=264)	Comparable annualized relapse rate of DMF to FTY (rate ratio 1.45 (0.53-3.99)). Comparable MRI activity of DMF to FTY (odds ratio 1.38 (0.83-2.32))	DMF patients discontinued therapy earlier compared to FTY (odds ratio 1.98 (1.18-3.23))
Vollmer et al., 2018 [38]	n=1272 (DMF n=737, FTY=535)	Comparable relapse rate of DMF to FTY (OR 1.27 (0.90-1.80)). Comparable MRI activity of DMF to FTY (OR 1.13 (0.83-1.55))	DMF patients discontinued therapy earlier compared to FTY (odds ratio 1.55 (1.21-1.99))
Laplaud et al., 2019 [39]	n=1770 (DMF n=1057, FTY 713)	Comparable relapse rate after one and two years. The adjusted proportion of patients with at least one new T2 lesion after two years was lower in DMF versus FTY (60.8% vs 72.2%, odds ratio 0.60, P<0.001). Lack of effectiveness was reported for 8.5% of DMF-treated patients vs 14.5% of FTY-treated patients (OR 0.54, P<0.001)	Adverse events accounted for 16% of TRF-treated patients and 21% of DMF-treated patients after two years (OR 1.39, P<0.001)

Dosing and Pharmacokinetics

Dosing of MMF typically starts at 95 mg twice daily (BID) for seven days, followed by an increase to a maintenance dose of 190 mg BID. It can be taken with or without food and is swallowed whole and intact. No recommendations for dosage adjustment are present for those with body weight, age, or gender differences [28]. The median Tmax of MMF is 4.03 hours, and approximately 27-45% are bound to human plasma proteins [28]. Data from Kuchimanchi et al. also shows that in 341 healthy volunteers and 48 patients with MS, there were revealed to be an estimated MMF clearance, volume of distribution, and absorption rate constant (Ka) of 13.5 L/g, 30.4 L, and 5.04 h^-1^, respectively. MMF clearance increased with body weight and decreased with renal function. Ka was reduced in low-, medium-, and high-fat meals by 37%, 51%, and 67%, respectively, for MMF [33]. Approximately 60% of MMF is excreted via CO2 exhalation, with renal and fecal elimination accounting for 16% and 1% of the dose, respectively [28].

Comparison of MMF vs other DMT

MMF Efficacy and Tolerability

MMF has proven efficacious and relatively safe in patients with relapsing forms of MS, including CIS, RRMS, and active SPMS. Data supporting its safety and efficacy profile can essentially be summed up by three main phase III studies: DEFINE, CONFIRM, and, their long-term extension study, ENDORSE [34-36]. Importantly, MMF (Bafiertam®), DMF (Tecfidera®), and DRF (Vumerity®) are oral bioequivalents, with MMF being the major active metabolite of both DMF and DRF. DMF was the first of the three drugs to be studied and receive FDA approval in 2013, and thus, the approval of DRF (in 2019) and MMF (in 2020) was based mainly upon bioequivalence, safety, and efficacy data for DMF [40-42]. As such, in outlining studies showing the favorable benefit-risk of DMF in RRMS patients, we demonstrate MMF's assumed efficacy and safety in RRMS patients. Table 1 summarizes the results and outcomes of clinical trials comparing DMF to PBO.

In the first pivotal phase III study, DEFINE, patients (n=1234) were randomly assigned to receive either DMF 240 mg BID, DMF 240 mg thrice daily (TID), or a PBO [34]. Patients were then followed on this regimen for two years, and their disease progression was analyzed. Study authors report that the effects of DMF were evident at 12 weeks and sustained after that. The estimated proportion of patients who experienced a relapse was significantly lower in those receiving DMF, BID, or TID than those receiving PBO (27% with DMF BID and 26% with DMF TID vs 46% with PBO). The annualized relapse rate (ARR, the total number of relapses divided by the number of patient-years in the study) at two years was 0.17 with DMF BID and 0.19 with DMF TID, compared to 0.36 with PBO. This represents a relative reduction of 53% and 48%, respectively, with the two DMF regimens. The proportion of patients with the progression of disability was also significantly lower in the DMF groups, estimated at 16% with DMF BID and 18% with DMF TID vs 27% with PBO. Finally, DMF also significantly reduced the number of new or enlarging T2-weighted hyperintense or gadolinium-enhancing lesions on MRI. In summary, the results of the DEFINE trial showed that in patients with RRMS, both DMF regimens significantly decreased the proportion who experienced a relapse, the ARR, the progression of disability, and the number of MS lesions on MRI compared to the PBO.

Regarding safety profile, the DEFINE study found that the overall incidence of adverse events (AEs) was similar across the three groups, with most being mild to moderate severity. AEs that occurred more frequently in the DMF treatment groups included flushing, GI events (e.g., diarrhea, nausea, vomiting, and abdominal pain), proteinuria, pruritis, elevated liver aminotransferase levels, and decreased lymphocyte counts. Flushing and GI events had the highest occurrence within the first month of treatment. The incidence of any serious adverse event (SAE) was also similar across the three study groups (21% with PBO, 18% with DMF BID, and 16% with DMF TID), with MS relapse being the most frequent. There was no increased risk of serious infections, opportunistic infections, or malignant neoplasms seen with DMF treatment. Patients receiving DMF were found to have decreases in mean white blood cell count and lymphocyte count during the first year (by approximately 10% and 28%, respectively), which then plateaued; mean values remained within normal limits. Finally, elevated liver aminotransferase levels were observed at a greater incidence in patients receiving DMF (6% of each group) compared to PBO (3%), mostly between months 1 and 6. That said, there were no reports of hepatic failure.

In the second of the pivotal phase III studies, CONFIRM, patients (n=1417) were randomly assigned to receive either DMF 240 mg BID, DMF 240 mg TID, glatiramer acetate (GA) 20 mg once daily (QD), or PBO [35]. GA is an active agent included in the study as a reference comparator; however, the study did not aim to test the efficacy of DMF vs GA. Patients followed their assigned regimen for two years as in the DEFINE trial. The ARR at two years was significantly lower with DMF or GA compared to PBO (0.22 with DMF BID, 0.20 with DMF TID, and 0.29 with GA vs 0.40 with PBO). Respectively, this represents relative reductions of 44%, 51%, and 29% compared to PBO. The estimated proportion of patients with relapse in two years was 41% in the PBO group compared to 29%, 24%, and 32% in the DMF BID, DMF TID, and GA groups, respectively. Compared to the PBO, all three treatment regimens significantly reduced mean numbers of new or enlarging T2-weighted hyperintense lesions or T1-weighted hypointense lesions at two years.

Interestingly, the CONFIRM trial did not find significant reductions in disability progression with DMF or GA compared to PBO. This contrasts with the results of the DEFINE trial; however, one may attribute this difference to the fact that in the CONFIRM trial, the proportion of patients with disability progression in the PBO group was only 17%, which was markedly less than that in the DEFINE trial (27%) [34,35]. In summary, the efficacy results of the CONFIRM trial were largely consistent with previous DMF studies and the DEFINE trial. Namely, in patients with RRMS, DMF treatment regimens (regardless of BID or TID dosing) significantly reduced the proportion of patients who had a relapse, the ARR, the disability progression, and the number of MS lesions on MRI compared to PBO.

Accompanying these efficacy findings were similar incidences of AEs and SAEs across all four study groups in the CONFIRM trial. Flushing and GI events of mild or moderate severity were once again the most common AEs associated with DMF treatment, and the incidence of these decreased with time. There was no increased risk of serious infections, opportunistic infections, or malignant neoplasms seen with DMF treatment. Decreases in mean white blood cell count and lymphocyte count (by approximately 12% and 30%, respectively) were found during the first year of DMF treatment; however, values then plateaued and remained within normal limits. Interestingly, elevations in liver aminotransferases occurred at a similar incidence in all treatment groups, which counters that observed in the DEFINE study (greater incidence of elevations in the DMF treatment groups). In general, however, the observed safety profile of DMF was similar to that of the CONFIRM and DEFINE studies.

The ENDORSE trial is the last of the three major trials investigating the safety and efficacy of DMF in the treatment of relapsing MS. This was a long-term extension of the DEFINE and CONFIRM trials in which enrolled patients (n=1736) were followed for a combined total of up to 13 years while receiving DMF treatment [42]. The median combined follow-up period was 8.76 years. Patients randomized to DMF 240 mg BID or TID in DEFINE/CONFIRM continued the same dose at the start of ENDORSE, whereas PBO or GA (CONFIRM-only) patients were re-randomized 1:1 to DMF BID or TID. Upon the FDA's approval of the DMF BID regimen in 2013, patients receiving DMF TID were switched to DMF BID at their next study visit. For patients who received continuous treatment with DMF BID (DMF/DMF, n=501), the overall ARR remained consistent and low at 0.143. For patients with a delayed start to DMF treatment (PBO/DMF), the ARR decreased after initiating DMF and remained low after that (ARR 0.33 for years 0-2 vs 0.15 for years 3-10). The study applied the Expanded Disability Status Scale (EDSS) as a measure of disease worsening taken every 24 weeks. They found that, over 10 years, there was no 24-week confirmed disability worsening for 72% of DMF/DMF patients and 73% of PBO/DMF patients.

Regarding safety profile, ENDORSE found an overall similar incidence of AEs and SAEs as recorded in DEFINE, CONFIRM, and real-world data sets [34-36]. Most AEs were reported as mild to moderate severity, the most common being MS relapse and nasopharyngitis, occurring in 39% and 26%, respectively [36]. The most common SAEs were MS relapse and fall. Around 14% of patients discontinued the treatment because of AEs (4% GI disorder). The risk of infection (including herpes zoster), serious infection, hepatic and renal disorders, and malignant neoplasms remained low with long-term treatment of DMF. That said, there was a case of PML in the setting of prolonged, severe lymphopenia in a patient receiving DMF [36,43]. The patient's lymphopenia developed 12 months after the initiation of DMF (lymphocyte count ranged from 290 to 580 cells per cubic millimeter) and persisted for 3.5 years [43]. Following clinical deterioration and unresponsiveness to first-line treatments for MS exacerbation, the patient was diagnosed with PML after 4.5 years of DMF treatment (based on MRI findings and a polymerase chain reaction (PCR) positive for JC virus in the cerebrospinal fluid). The patient died within two months of their clinical decline. This resulted in updated prescribing information and risk mitigation strategies, including absolute lymphocyte count (ALC) discontinuation criteria. According to US DMF label guidance, clinicians should consider the interruption of DMF treatment if ALCs <0.5x109/L persist for >6 months [36-42,44,45]. Lymphopenia is the strongest risk factor for DMF-associated PML. The overall incidence of prolonged, severe lymphopenia in all DMF-treated patients in the ENDORSE trial was minimal (2.8%). With that, prolonged, severe lymphopenia significantly rarely developed for the first time after year 3 of DMF treatment, occurring in just nine of 2263 patients (<1%). Importantly, the incidence of DMF-associated PML remains very rare in the post-marketing setting, with an estimated rate of 1.07 cases per 100,000 person-years of DMF exposure [36,45]. Overall, ENDORSE demonstrated the sustained efficacy and safety of DMF and its positive benefit-risk profile for the long-term treatment of RRMS.

Additional information regarding the safety and efficacy of DMF for MS patients comes from several post-marketing real-world studies and retrospective analyses [37-39,46-51]. One of these studies, PROTEC, investigated the effectiveness of DMF on disease activity and patient-reported outcomes (PROs) in patients with RRMS [46]. They found that at 12 months post-DMF initiation, unadjusted ARR was 75% lower (0.161 vs 0.643) overall and multiple PRO measures (including fatigue, treatment satisfaction, daily living, and work) showed improvement compared to pre-DMF. Overall, 88% of patients remained relapse-free 12 months after treatment.

Alroughani et al. prospectively assessed DMF's safety and efficacy and the occurrence of lymphopenia in a cohort of MS patients using the national MS registry [47]. Their findings were largely consistent with those of clinical trials, with an increased proportion of relapse-free patients (from 51.2% to 89.9%) and a decreased proportion of patients with disease activity on MRI (from 61.1% to 15.1%) after DMF exposure (mean exposure duration 20.5 months). Although this study did find a 34% decrease in mean ALCs, researchers concluded that ALC profiles in DMF-treated patients were generally stable throughout their observation period of 20 months and reported only three patients had severe lymphopenia requiring discontinuation of DMF (2.5%, consistent with clinical trials) [34,35].

MMF vs DMF vs DRF

As previously stated, MMF is the active metabolite of DMF and DRF. Studies looking at the comparative pharmacokinetics and bioavailability of MMF following a single oral dose of MMF (Bafiertam®) vs DRF (Vumerity®) vs DMF (Tecfidera®) found that, at their respective therapeutic doses, the medications can be considered bioequivalent [52,53]. The side effect profile of each of the three drugs is like that of DMF (described above); however, some studies have demonstrated that MMF and DRF may have a lesser frequency of GI AEs than DMF [5,6,54,55]. For example, Wynn et al. conducted a randomized, double-blind, five-week-long, phase I study investigating the GI tolerability of MMF vs DMF using a derivative of the self-administered Modified Overall Gastrointestinal Symptom Scale (MOGISS) [6]. They found that, compared to DMF, MMF showed improved GI tolerability, less frequent and less severe GI symptoms, and lower discontinuation rates because of GI AEs. An analogous phase III study investigated the GI tolerability of DMF vs DRF in patients with RRMS using two self-administered GI symptoms scales [5]. The results of this study suggested that, compared to DMF, DRF similarly had improved GI tolerability, again with less frequent and less severe GI symptoms and lower discontinuation rates because of GI AEs. One proposed explanation for this finding is that compared to DMF, the metabolism of DRF leads to a lower concentration of methanol in the small intestine and, thus, fewer GI side effects. One may reasonably infer that this improved GI profile of DRF and MMF compared to DMF is associated with a positive impact on quality of life and further increased likelihood of treatment persistence and medication adherence in patients with relapsing forms of MS [56,57].

A final notable topic of discussion is hepatotoxicity secondary to treatment with a fumarate medication. While mild to moderate elevations in liver aminotransferases were observed in clinical trials on DMF, the elevations were usually transient and not associated with symptoms of liver injury [34-36]. In other words, there were no reported cases of acute hepatitis or clinically apparent liver injury with jaundice from the DMF clinical trials. Despite this, several cases have been reported since its approval and more widespread use [54]. All patients recovered upon discontinuing DMF, and there have been no reports of chronic injury or hepatic failure. There have been no reported cases of clinically apparent liver injury with DRF or MMF, but clinical experience with these drugs has been limited [54,5,55]. Given their tolerability profiles similar to DMF, all three fumarates are suspected to be rare causes of clinically apparent liver injury. Taken together, the National Institutes of Health (NIH) LiverTox has assigned DMF a likelihood score of C (probably rare cause of clinically apparent liver injury) and MMF and DRF a likelihood score of E (unproven but suspected rare cause of clinically apparent liver injury) (Table 3) [54].

**Table 3:** Summarizing fumarate characteristics MMF: monomethyl fumarate; DRF: diroximel fumarate; DMF: dimethyl fumarate

Medication	Gastrointestinal side effects	Hepatotoxicity
MMF (Bafiertam®)	Lesser frequency of gastrointestinal adverse events compared to DMF	Likelihood score: E (unproven but suspected rare cause of clinically apparent liver injury)
DRF (Vumerity®)	Improved gastrointestinal tolerability, less frequent and less severe symptoms, and lower discontinuation rates compared to DMF	Likelihood score: E (unproven but suspected rare cause of clinically apparent liver injury)
DMF (Tecfidera®)	Common gastrointestinal adverse effects	Likelihood score: C (probably rare cause of clinically apparent liver injury)

Discussion

MMF has proven efficacious and relatively safe in patients with relapsing forms of MS, including CIS, RRMS, and active SPMS. Data supporting this largely comes from three pivotal phase III studies, DEFINE, CONFIRM, and ENDORSE, performed on DMF, a prodrug of MMF. More specifically, DEFINE and CONFIRM demonstrated how DMF significantly reduced the proportion of patients who had a relapse, the ARR, the disability progression, and the number of MS lesions on MRI compared to PBO, without a significantly increased incidence of AEs. ENDORSE, the long-term extension study, demonstrated the sustained efficacy and safety of DMF and its positive benefit-risk profile for the long-term treatment of relapsing forms of MS. Furthermore, in direct comparison to other fumarates, there is considerable evidence indicating that MMF (as well as DRF) demonstrates less frequent GI AEs compared to DMF. Besides the direct improvement in side effect profile, this difference has been associated with increased quality of life as well as increased likelihood of medication adherence in patients who require pharmacotherapy for relapsing forms of MS. Additionally, MMF was classified in the lowest risk category (E) for probability of causing clinically apparent liver injury, meaning that it is considered to be an unproven but suspected rare cause; in contrast, DMF was assigned a likelihood score of C, labeling it as a probably rare cause of clinically apparent liver injury.

## Conclusions

The currently available data surrounding the use of MMF in patients with relapsing forms of MS strongly support its safety and efficacy, with additional benefits in terms of adverse side effects when compared to its prodrug DMF. Still, as this formulation is relatively new, an unavoidable lack of data remains in determining long-term outcomes and complications, specifically of MMF as its own formulation as opposed to the bioequivalent metabolite to the older DMF, for which more information and data are available. Additional research should be conducted to solidify the reliability of these indications from currently available studies and obtain more evidence of rare or delayed side effects of MMF.
